# Histidine‐rich glycoprotein augments natural killer cell function by modulating PD‐1 expression via CLEC‐1B

**DOI:** 10.1002/prp2.481

**Published:** 2019-05-22

**Authors:** Yoshito Nishimura, Hidenori Wake, Kiyoshi Teshigawara, Dengli Wang, Masakiyo Sakaguchi, Fumio Otsuka, Masahiro Nishibori

**Affiliations:** ^1^ Department of Pharmacology Okayama University Graduate School of Medicine, Dentistry and Pharmaceutical Sciences Okayama Japan; ^2^ Department of Cell Biology Okayama University Graduate School of Medicine, Dentistry and Pharmaceutical Sciences Okayama Japan; ^3^ Department of General Medicine Okayama University Graduate School of Medicine, Dentistry and Pharmaceutical Sciences Okayama Japan

**Keywords:** C‐type lectin‐like receptor, histidine‐rich glycoprotein, natural killer cell, programmed‐death‐1

## Abstract

Augmentation of natural killer (NK) cell cytotoxicity is one of the greatest challenges for cancer immunotherapy. Although histidine‐rich glycoprotein (HRG), a 75‐kDa glycoprotein with various immunomodulatory activities, reportedly elicits antitumor immunity, its effect on NK cell cytotoxicity is unclear. We assessed NK cell cytotoxicity against K562 cells. We also measured concentrations of cytokines and granzyme B in the cell supernatant. The proportion of CD56^bright^ NK cells and NK cell surface PD‐1 expression was assessed with flow cytometry. The neutralizing effects of anti‐C‐type lectin‐like receptor (CLEC) 1B against HRG were also measured. NK cell morphological changes were analyzed via confocal microscopy. HRG significantly increased NK cell cytotoxicity against K562 cell lines. HRG also increased the release of granzyme B and the proportion of CD56^bright^ NK cells. Further, HRG was able to decrease NK cell surface PD‐1 expression. The effects of HRG on NK cells were reversed with anti‐CLEC‐1B antibodies. Additionally, we confirmed NK cell nuclear morphology and F‐actin distribution, which are involved in the regulation of cytotoxic granule secretion. Because both PD‐1 and CLEC‐1B are associated with prognosis during malignancy, HRG incorporates these molecules to exert the antitumor immunity role. These facts indicate the potential of HRG to be a new target for cancer immunotherapy.

**Abbreviations**
CLEC: C‐type lectin‐like receptor
HRG: Histidine‐rich glycoprotein
HSA: Human serum albumin
PD‐1: Programmed‐death‐1
RANTES: Regulated on activation, normal T‐cell expressed and secreted

## 1 INTRODUCTION

One challenge of cancer immunotherapy is understanding the complex regulatory interactions between effector and target cells. Natural killer (NK) cells are unique innate lymphocytes that target viral infections and tumor cells. Their functions as effector cells include cytokine production, such as interferon‐gamma (IFN‐γ), exocytosis of cytotoxic granules containing granzyme B, and direct killing of target cells through Fas ligands and tumor necrosis factor (TNF)‐related apoptosis‐inducing ligand pathways.^1^, ^2^, ^3^, ^4^, ^5^, ^6^ NK cells also produce chemokines including RANTES (regulated on activation, normal T‐cell expressed and secreted) that induce T‐cell migration and collaboratively kill target cells.^7^ A recent study found that programmed‐death‐1 (PD‐1), an inhibitory checkpoint molecule, is expressed on NK cells. Given this, the regulation of PD‐1 expression on NK cells might provide therapeutic potential for antitumor immunotherapy.^3^

Histidine‐rich glycoprotein (HRG) is a 75‐kDa glycoprotein produced by the liver. Plasma levels of HRG are maintained at approximately 1 μmol/L, with a relatively rapid turnover.^8^, ^9^ Because of its multidomain, three‐dimensional structure, HRG binds a variety of molecules such as heparin, fibrinogen, and heme,^9^ which facilitate numerous modulatory activities, such as cell adhesion, angiogenesis, and thrombosis.^10^, ^11^, ^12^ Researchers have hypothesized that decreased plasma levels of HRG are responsible for the development of pulmonary fibrosis, preeclampsia, and sepsis.^13^, ^14^ In septic pathophysiology, the rapid decrease in plasma HRG might diminish its regulatory effects on neutrophils, vascular endothelial cells, and erythrocytes, leading to impaired passage of neutrophils through capillary vessels. It may also cause injury to vascular endothelial cells via the production of reactive oxygen species by adhered neutrophils and the formation of immune‐thrombosis.^13^, ^14^, ^15^, ^16^, ^17^, ^18^, ^19^ Thus, physiological concentrations of HRG in plasma might maintain the quiescence of circulating neutrophils preventing the uncontrolled activation of vascular endothelial cells and thrombus formation.^18^ Moreover, plasma HRG is markedly decreased in septic patients in intensive care units, and it is superior to procalcitonin and presepsin as a biomarker in predicting sepsis.^14^ In addition, HRG might potentiate the antitumor effects of tumor‐associated macrophages via antiangiogenic effects.^20^, ^21^

Some researchers suggest that NK cell‐mediated cytotoxicity is augmented by various therapeutic agents and peptides.^22^, ^23^, ^24^, ^25^, ^26^, ^27^, ^28^, ^29^, ^30^ Among them, lenalidomide, an immunomodulatory drug for multiple myeloma, appears to indirectly restore NK cell function by inducing IFN‐γ secretion, facilitating actin remodeling, and modulating PD‐1/PD‐L1 interactions.^31^, ^32^ HRG inhibits tumor growth by inducing the migration of macrophages, cytotoxic T‐cells (CTLs), and NK cells through the downregulation of placental growth factor.^33^ However, receptors of HRG and the exact mechanisms associated with the effects of HRG on NK cell cytotoxicity have not yet been elucidated.

C‐type lectin‐like receptors (CLECs) comprise a diverse family of transmembrane pattern recognition receptors expressed primarily on myeloid cells.^34^ Their functions include intercellular communication, host defense against infection, cancer immunity, and the maintenance of different homeostatic responses.^35^ CLEC‐1A is a representative CLEC, and its encoding gene is closely linked to the NK gene complex.^34^ NK cells express a variety of functional CLEC superfamily receptors.^36^ Notably, CLEC‐1B, an immunoreceptor tyrosine‐based activation motif (ITAM)‐containing receptor, formerly known as CLEC‐2, has been extensively researched based on its immunomodulatory functions. CLEC‐1B is linked to inflammation during sepsis, thrombosis, and cancer immunity.^37^, ^38^, ^39^, ^40^ Wang et al reported that CLEC‐1B might suppress AKT signaling and cancer cell invasiveness.^40^ More recently, it was suggested that CLEC‐1B expression levels can predict outcome for certain cancers.^37^ These reports make CLEC‐1B a fascinating therapeutic target when considering antitumor immunity.

Here, we hypothesized that the inhibitory effects of HRG on tumors could be attributed to its direct immunomodulatory effects on NK cells through the augmentation of cytokine production and modulation of PD‐1 expression. We also anticipated that CLEC, especially CLEC‐1B, might be a potential receptor for HRG. The purpose of this study was to examine whether HRG enhances NK cell cytotoxicity and regulates cytokine production by these cells through the stimulation of specific receptors, focusing on revealing molecular and pathological associations with antitumor immunity.

## 2 MATERIALS AND METHODS

### 2.1 Antibodies and reagents

FITC‐labeled anti‐human CD279 (PD‐1; EH12.2H7), PE‐labeled anti‐human CD56 (HCD56), PerCP‐labeled anti‐human CD3 (SK7), and PE‐labeled Mouse IgG1κ Isotype control (MOPC‐21) antibodies were purchased from BioLegend (San Diego, CA). Anti‐human CLEC‐1 goat polyclonal, anti‐human CLEC‐2 goat polyclonal, and goat polyclonal control IgG antibodies were purchased from R&D Systems (Minneapolis, MN). Phalloidin‐Alexa Fluor 568, deoxyribonuclease I‐Alexa Fluor 488, Hoechst33342, and Calcein‐AM were obtained from Thermo Fisher Scientific (Waltham, MA). Recombinant human IL‐2 was purchased from Sigma‐Aldrich (St. Louis, MO). For NK cell incubation, IL‐2 concentrations were adjusted to 200 U/mL in RPMI‐1640 medium.

### 2.2 Cell culture

K562 cells were obtained from RIKEN BioResource Research Center Cell Bank (Ibaraki, Japan). The cells were cultured in Dulbecco's modified Eagle's medium with 10% fetal calf serum, L‐glutamine, and penicillin and streptomycin, and maintained in a humidified 5% CO_2_ environment at 37°C.

### 2.3 Isolation of peripheral blood mononuclear and NK cells from human blood

Peripheral blood mononuclear cells (PBMCs) were purified from healthy donors using Lymphoprep density gradient media (Alere Technologies AS, Oslo, Norway). In accordance with the ethical approval guidelines of Okayama University, written informed consent was obtained from healthy volunteers (n = 5), and blood was drawn from the cubital vein. Human primary NK cells were isolated using the EasySep Human NK Cell Enrichment Kit in accordance with the manufacturer's protocol (STEMCELL Technologies, Vancouver, Canada). The purity of NK cells was confirmed to be greater than 90% by flow cytometric analysis using anti‐CD3 and anti‐CD56 monoclonal antibodies (data not shown).

### 2.4 HRG purification from human plasma

HRG was purified from fresh frozen plasma samples obtained from the Japanese Red Cross Society as previously described.^41^

### 2.5 Cytotoxicity assays

NK cell‐mediated cytotoxicity was measured using a lactate dehydrogenase (LDH) Cytotoxicity Detection Kit (Takara Bio, Shiga, Japan). The extent of cytotoxicity was calculated according to the manufacturer's instructions. To establish the appropriate effector‐to‐target (E:T) ratios, K562 cells and PBMCs were incubated for 6 hours with different cell concentrations. Next, K562 cells were incubated with either PBMCs at an 80:1 E:T ratio or NK cells at a 10:1 E:T ratio for 6 hours with 5% CO_2_ at 37°C. NK cells were used after overnight incubation in RPMI‐1640 medium with IL‐2. After this, cells were washed three times to completely remove IL‐2. Then, cells were suspended in RPMI1640 without phenol red and 1 μmol/L HRG, 1 μmol/L human serum albumin (HSA), or Hank's balanced salt solution (HBSS) were added to the mixtures as test compounds. After incubation, the tubes were centrifuged at 500***g*** for 3 minutes. The supernatant was then used for the determination of LDH. Percent NK cell‐mediated cytotoxicity was presented based on the following equation: % cytotoxicity = (experimental value − effector cell spontaneous LDH release − K562 spontaneous LDH release)/(K562 maximum LDH release − K562 spontaneous LDH release) × 100. A background control value was subtracted from all values.

### 2.6 Cytometric bead analysis (CBA)

Concentrations of IL‐2, IFN‐γ, granzyme B, and RANTES in the cell supernatant were measured using Flex CBA kits according to the manufacturer's instructions (BD Biosciences, Franklin Lakes, NJ). In brief, isolated human NK cells were cultured in RPMI 1640 with or without IL‐2 overnight. NK cells and K562 cells were cocultured in RPMI1640 without phenol red with a 10:1 E:T ratio and 1 μmol/L HRG, 1 μmol/L HSA, or HBSS for 16 hours. Supernatants were then acquired for analysis after centrifugation at 500***g*** for 3 minutes.

### 2.7 CD56^bright^ and CD56^dim^ NK cell identification by flow cytometry

NK cells were stained with a PE‐labeled anti‐CD56 monoclonal antibody and separated into CD56^bright^ and CD56^dim^ groups by flow cytometry. Briefly, NK cells were cultured overnight with 5% CO_2_ at 37°C after purification with or without IL‐2. The cells were then cocultured with K562 cells at a 10:1 E:T ratio in RPMI1640 without phenol red. After 1 hour of incubation, CD56 expression on NK cells was analyzed using a MACSQuant Analyzer and MACSQuantify Software 2.11 (Miltenyi Biotec, Bergisch Gladbach, Germany).

### 2.8 Cell surface PD‐1 expression analysis

To elucidate the effects of HRG on PD‐1 expression on NK cells, these cells were cultured overnight with 5% CO_2_ at 37°C after purification with or without IL‐2. NK cells were incubated with K562 cells in RPMI1640 without phenol red at a 10:1 E:T ratio in the presence of 1 μmol/L HRG, 1 μmol/L HSA, or HBSS for 4 hours. The cells were then stained with FITC‐labeled anti‐PD‐1 and PE‐labeled anti‐CD56 antibodies for flow cytometric analysis using a MACSQuant Analyzer.

### 2.9 Effects of anti‐CLEC antibodies on the effect of HRG immunomodulation

Anti‐CLEC‐1A, anti‐CLEC‐1B polyclonal, and goat IgG control antibodies were added to each group subjected to PD‐1 analysis. The analysis of NK cell surface PD‐1 expression was performed as described for each method. For this, 0.5 μmol/L HRG or 0.5 μmol/L HSA were added to each group. Anti‐CLEC and control antibodies were added at a concentration of 10 μg/mL before coculture.

### 2.10 Observation of NK cell morphological changes

To clarify the effects of HRG on NK cell morphology, the cells were incubated with HRG, HSA, or HBSS at 1 μmol/L for 4 hours after overnight stimulation with IL‐2‐containing RPMI1640. Cell shape was observed by calcein staining as described previously.^18^ An IN Cell Analyzer 2000 System (GE Healthcare, Little Chalfont, UK) was used for observation. The data were analyzed using IN Cell Investigator Version 1.62 (GE Healthcare, Little Chalfont, UK).

### 2.11 Observation of F‐actin/G‐actin distribution in NK cells

After overnight incubation with IL‐2, NK cells were cocultured with K562 at a 10:1 E:T ratio in the presence of HRG, HSA, or HBSS at 1 μmol/L for 4 hours in RPMI1640. The cell suspensions were then gelatinized using Smear Gell (GenoStaff, Tokyo, Japan) on amino‐propyltriethoxy silane (APS)‐coated glass slides (Matsunami, Tokyo, Japan) according to the manufacturers’ instructions. Cells were then fixed with 4% paraformaldehyde. After fixation, the cells were treated with 0.1% triton‐X‐100 for membrane permeabilization, followed by staining with Phalloidin‐Alexa568 (F‐actin), DNase I‐Alexa488 (G‐actin), and DAPI (nuclei). Samples were observed with a confocal microscope (LSM780, Carl Zeiss, Oberkochen, Germany).

### 2.12 Statistical analysis

Tukey‐Kramer or one‐way ANOVA tests were performed. Data are presented as mean values ± SEM. *P*‐values <0.05 were considered statistically significant. All statistical analyses were conducted using JMP Version 13 (SAS Institute, Cary, NC).

## 3 RESULTS

### 3.1 HRG augments NK cell‐mediated cytotoxicity toward K562 cells

**Figure 1:**
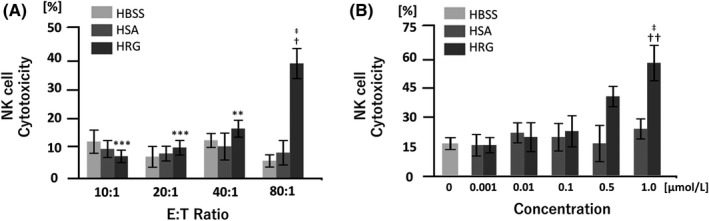
Histidine‐rich glycoprotein (HRG) augments natural killer (NK) cell‐mediated cytotoxicity toward K562 cells. Peripheral blood mononuclear cells (PBMCs) or NK cells purified from the peripheral blood of healthy volunteers were incubated with K562 cells. Cytotoxicity was evaluated by measuring the extent of lactate dehydrogenase (LDH) release after the indicated incubation periods. (A) Cytotoxicity experiments were performed by incubating PBMCs with K562 cells in RPMI1640 without phenol red at different effector‐target (E:T) ratios for 6 hours in the presence of HRG (1 μmol/L), human serum albumin (HSA; 1 μmol/L), or control (Hank's balanced saline solution; HBSS). The results are the mean ± SEM of three determinations. †*P* < 0.05 compared to HBSS. (B) Purified NK cells were incubated with K562 cells at a 10:1 E:T ratio for 6 hours in the presence of different concentrations of HRG, HSA (1 μmol/L, 0.1 μmol/L, 0.01 μmol/L, 0.00 1 μmol/L), or HBSS (0 μmol/L). The results are the mean ± SEM of four or six determinations. **P < 0.01, ***P < 0.001 compared to HRG at an E:T ratio of 80:1. †*P* < 0.05, ††*P* < 0.01 compared to HBSS. ‡*P* < 0.05 compared to HSA

Cytotoxicity peaked with an 80:1 E:T ratio (Figure 1A). After confirming the appropriate E:T ratio, NK cell cytotoxicity was assessed quantitatively by measuring LDH release in the supernatants of NK and K562 cell cocultures. To examine whether NK cell cytotoxicity was augmented in the presence of HRG, we first compared the cytotoxicity of PBMCs against K562 cells. Based on these results, 1 μmol/L HRG significantly increased NK cell‐mediated cytotoxicity compared to HBSS and HSA groups (Figure 1A). Next, we cocultured purified NK cells with K562 cells to rule out the possibility that HRG was effecting PBMCs other than NK cells. We observed a similar dose‐dependent increase in cytotoxicity; a significant effect was noted with a concentration of 1 μmol/L. Incubation with HBSS and HSA did not produce any effects (Figure 1B).

### 3.2 Effects of HRG on cytokine production and granzyme B secretion from NK cells

**Figure 2:**
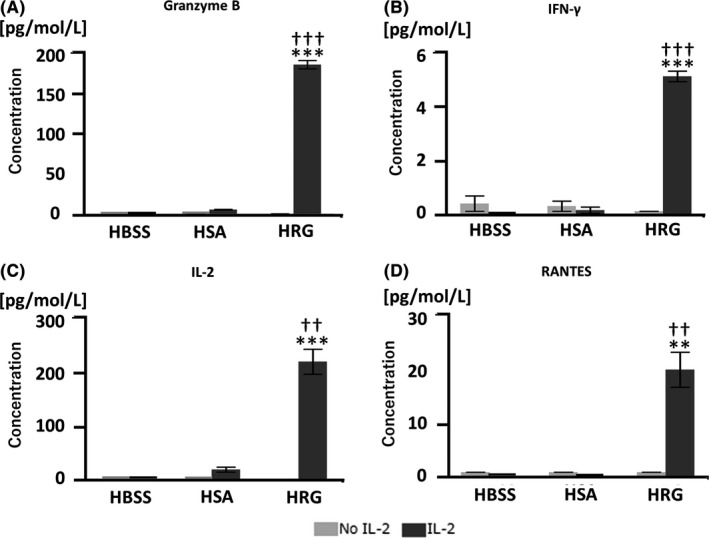
Histidine‐rich glycoprotein (HRG) increases the secretion of granzyme B and cytokines from natural killer (NK) cells. Purified NK cells were pretreated in the presence or absence of IL‐2 overnight at 37°C. After extensive washing, NK cells were incubated with K562 cells in RPMI1640 without phenol red at an effector‐target (E:T) ratio of 10:1 for 16 hours at 37°C, supernatants were collected for determining granzyme B (A) and cytokine concentrations (B–D). The results are the mean ± SEM of five experiments (A) and three experiments (B‐D). **P < 0.01, ***P < 0.001 compared to Hank's balanced saline solution (HBSS). ††*P* < 0.01, ^†††^ *P* < 0.001 compared to human serum albumin (HSA)

To explore the mechanism through which HRG modulates NK cell cytotoxicity, we quantified the concentration of various cytokines in the supernatants of cocultures. For this, we cocultured freshly isolated, IL‐2‐activated NK cells with K562 cells for 16 hours to quantify granzyme B levels in the supernatants. HRG not only increased granzyme B secretion from NK cells but also stimulated the production and release of IL‐2, IFN‐γ, and RANTES (Figure 2). Collectively, these results suggested that HRG could augment NK cell cytotoxicity through two different pathways, specifically direct toxicity and an indirect immunomodulatory effect via cytokine and chemokine production.

### 3.3 HRG increases the proportion of CD56^bright^ NK cells

**Figure 3:**
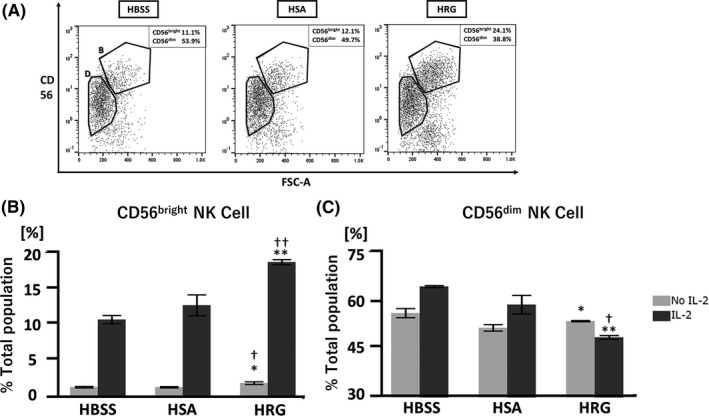
Histidine‐rich glycoprotein (HRG) increases the proportion of CD56^bright^ natural killer (NK) cells relative to CD56^dim^ NK cells. Purified NK cells were pretreated in the presence or absence of IL‐2 overnight at 37°C. After extensive washing, NK cells were incubated with K562 cells in RPMI1640 without phenol red at an effector‐target (E:T) ratio of 10:1 for 1 hour in the presence of HRG (1 μmol/L), human serum albumin (HSA; 1 μmol/L), or Hank's balanced saline solution (HBSS). After cells were stained with a PE‐labeled anti‐human CD56 antibody, flow cytometric analysis was performed with a MACSQuant Analyzer. (A) Representative images of the flow cytometric analysis of CD56 expression on NK cells. CD56^bright^ and CD56^dim^ cell populations were circled and indicated with bold “B” and “D”, respectively. (B) Percentages of CD56^bright^ NK cells and (C) CD56^dim^ NK cells were evaluated by flow cytometric analysis. The results are the mean ± SEM of three experiments. *P < 0.05, **P < 0.01 compared to HBSS. †*P* < 0.05, ††*P* < 0.01 compared to HSA

NK cells exist as three subpopulations, specifically CD56^bright^, CD56^dim^, and CD56^negative^ cells. Among them, CD56^bright^ NK cells are considered potential cytokine‐producing cells that play roles in various processes, including antitumor immunity.^42^ They also exert cytotoxic effects upon activation. In contrast, CD56^dim^ NK cells mainly exert cytotoxic effects independent of cytokine production.^43^ Therefore, we speculated that HRG might not only activate NK cells but also induce the differentiation of CD56^dim^ cells into CD56^bright^ cells. As expected, HRG significantly increased the population of CD56^bright^ NK cells after coculture with K562 cells (Figure 3A‐C). The effects of HRG on promoting differentiation into CD56^bright^ cells were also significant in groups not pretreated with IL‐2 (Figure 3B). Consistent with the increase in the CD56^bright^ cell population, the percentage of CD56^dim^ cells decreased in the HRG‐treated group (Figure 3C).

### 3.4 PD‐1 expression is decreased by HRG treatment

**Figure 4:**
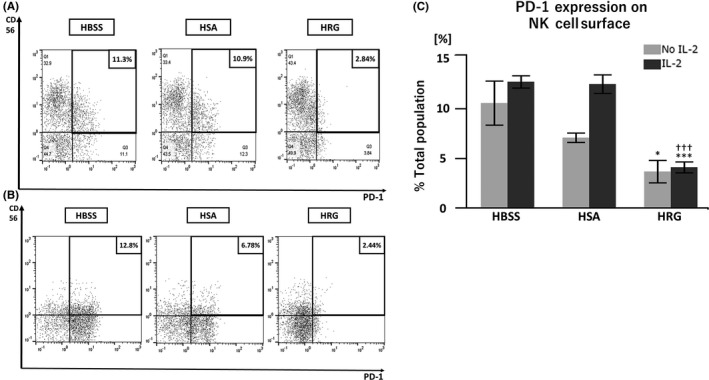
PD‐1 expression on natural killer (NK) cell surfaces is decreased after histidine‐rich glycoprotein (HRG) treatment. Purified NK cells were pretreated in the presence or absence of IL‐2 overnight at 37°C. After extensive washing, NK cells were incubated with K562 cells in RPMI1640 without phenol red at an effector‐target (E:T) ratio of 10:1 for 4 hours in the presence of HRG (1 μmol/L), human serum albumin (HSA; 1 μmol/L), or Hank's balanced saline solutions (HBSS). After cells were stained with FITC‐labeled anti‐human PD‐1 and PE‐labeled anti‐human CD56 antibodies, flow cytometric analysis was performed with a MACSQuant Analyzer. (A) Representative images of the flow cytometric analysis of IL‐2 pretreated cells are shown to illustrate PD‐1 expression on NK cells. (B) Representative images of the flow cytometric analysis of cells without IL‐2 pretreatment. (C) PD‐1 expression on NK cells was determined by flow cytometric analysis. The results are the mean ± SEM of three experiments. *P < 0.05, ***P < 0.001 compared to HBSS. ††† p < 0.001 compared to HSA

Because the extent of PD‐1 expression on the NK cell surface may be related to their cytotoxic activity, we performed flow cytometric analysis to quantify the PD‐1 expression level on NK cells. HRG also decreased NK cell surface PD‐1 expression, irrespective of IL‐2 pretreatment (Figure 4A‐C).

### 3.5 An anti‐CLEC‐1B polyclonal antibody inhibits the effects of HRG on NK cell PD‐1 expression

**Figure 5:**
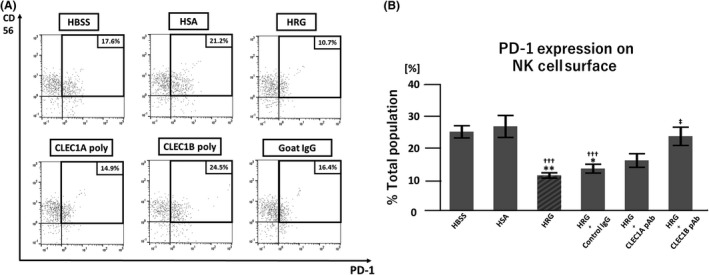
An anti‐CLEC1B polyclonal antibody inhibits the effect of histidine‐rich glycoprotein (HRG) on natural killer (NK) cell surface PD‐1 expression. IL‐2 pretreated NK cells were incubated with K562 cells in RPMI1640 without phenol red in the presence of HRG (0.5 μmol/L), human serum albumin (HSA; 0.5 μmol/L), or Hank's balanced saline solution (HBSS). To evaluate the inhibitory effect of anti‐CLEC antibodies, anti‐CLEC‐1A and anti‐CLEC‐1B human goat polyclonal antibodies (pAbs) were added to HRG groups (HRG + anti‐CLEC‐1A pAb or anti‐CLEC‐1B pAb). The antibody concentration was 10 μg/mL for each. (A) Representative images of flow cytometric analysis to evaluate PD‐1 expression on NK cells in the presence of HRG (0.5 μmol/L) with or without anti‐CLEC‐1A or anti‐CLEC‐1B pAbs. (B) The percentage of PD‐1‐positive cells among NK cells was determined by flow cytometric analysis of each group. The results are the mean ± SEM of nine experiments. *P < 0.05, **P < 0.01 compared to HBSS. †††*P* < 0.001 compared to HSA. ‡*P* < 0.05 compared to HRG+CLEC‐1B pAb

Next, we hypothesized that CLEC‐1B, a C‐type lectin‐like receptor that is expressed on NK cells, could be a HRG receptor on NK cells based on previous reports suggesting a relationship between CLEC‐1B and tumor immunity.^35^ Due to an abundance of potential epitopes, we used human anti‐CLEC polyclonal antibodies to examine the effect of HRG on NK cell PD‐1 expression. Anti‐CLEC antibodies were used at 10 μg/mL to minimize the immunomodulatory effects of the antibodies themselves. We set out to determine if the anti‐CLEC‐1B antibody could inhibit the effects of HRG on PD‐1 expression by flow cytometric analysis. As shown in Figure 5A,B, the anti‐CLEC‐1B antibody significantly inhibited this effect by suppressing cell surface PD‐1 expression, whereas the effect of anti‐CLEC‐1A was not significant.

### 3.6 HRG induces morphological changes associated with compact nuclear shapes and actin distribution in NK cells

**Figure 6:**
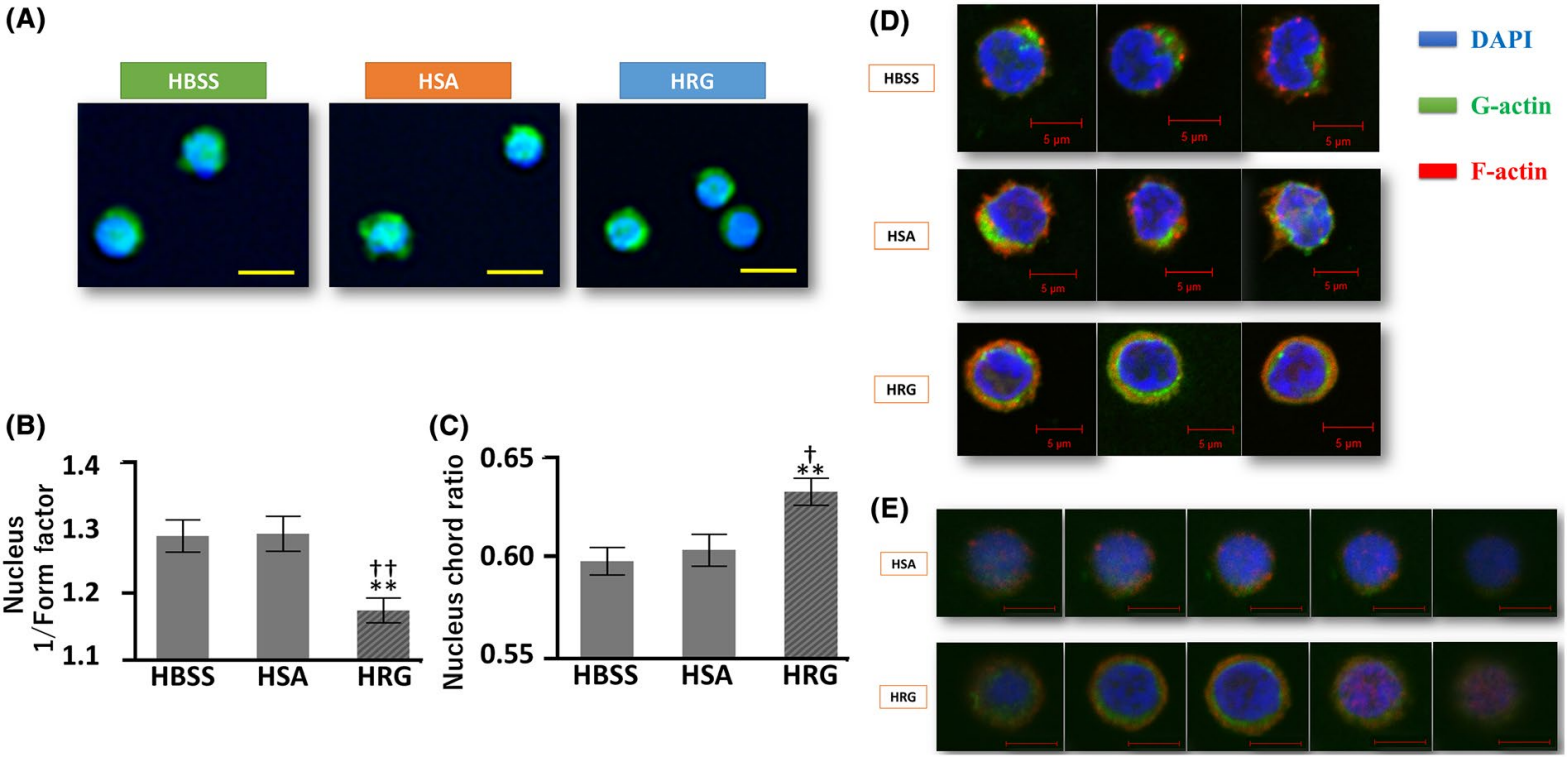
Histidine‐rich glycoprotein (HRG) induces natural killer (NK) cell nuclear morphological transformation and intracellular actin reorganization. Live cell morphological changes were analyzed using the In Cell Analyzer 2000 system. NK cell F‐actin/G‐actin distribution was analyzed by fluorescence staining and confocal microscopy (LSM780, Carl Zeiss, Oberkochen, Germany). All images were acquired at 63 × original magnification by confocal laser microscope at room temperature. Images were analyzed with ZEN lite 2012 64‐bit version Black edition (Carl Zeiss, Oberkochen, Germany). (A) Representative NK cell morphologies are shown after treatment with HRG (1 μmol/L), human serum albumin (HSA; 1 μmol/L), or Hank's balanced saline solution (HBSS) for 4 hours at 37°C. (B) Nuclear morphologies were objectively evaluated. The form factor represents nuclear roundness, for which a form factor of 1 indicates a perfect circle. (C) Nuclear chord ratio represents the ratios of nuclear long to short axes, with a chord ratio closer to 1 implying perfect roundness. NK cell nuclei were significantly rounder in the HRG group. The results are the mean ± SEM of 21 experiments. Scale bars, 10 μm. **P < 0.01 vs HBSS. †*P* < 0.05, ††*P* < 0.01 vs HSA. (D) NK cell F‐actin/G‐actin distribution was analyzed by fluorescence staining and confocal microscopy. (E) Representative optical sections of Z‐stack images of NK cells treated with HRG or HSA. F‐actin; Phalloidin‐Alexa Fluor 568 (Red), G‐actin; DNase I‐Alexa Fluor 488 (Green), nuclei; DAPI (Blue). Representative images are shown. Scale bars, 5 μm

Finally, based on a previous report that HRG induces morphological changes in neutrophils, to a more spherical shape, which were found to be associated with functional alterations,^18^ we speculated that similar effects could occur with NK cells. In addition, because the nuclear shape is associated with mechanical forces exerted by the cytoskeleton and because NK cell lytic granules are transported along F‐actin fibers,^44^ we utilized a live cell imaging device and confocal microscopy to visualize morphological changes. In HRG groups, NK cell nuclei became significantly rounder compared to those in HBSS and HSA groups (Figure 6A‐C). Based on fluorescent confocal microscopy analysis, we determined that F‐actin staining was localized mainly to the plasma membrane in a circular pattern in the HRG group. In contrast, F‐actin was observed as spots in HBSS and HSA groups (Figure 6D‐E).

## 4 DISCUSSION

Our data highlight the significance of HRG in augmenting NK cell cytotoxicity through several pathways in the following ways: (a) direct cytotoxicity via granzyme B release, (b) cytokine production and release, (c) an increase in the relative proportion of CD56^bright^ NK cells, (d) decreased expression of cell surface PD‐1, and (e) actin remodeling. Previous research suggested that HRG is related to antitumor immunity,^20^ but the exact mechanisms were unclear. Although CD56^bright^ and CD56^dim^ NK cells are both known to release cytokines and granzyme B, respectively, HRG not only increased the relative proportion of CD56^bright^ NK cells, but also enhanced granzyme B secretion. This suggests that HRG functions to augment cytotoxic granule production in CD56^dim^ cells, which primarily exert direct cytotoxic immunity,^43^ while facilitating the transition of CD56^dim^ to CD56^bright^ cells. Previously, it was reported that CD56^bright^ NK cells are increased in the tumor microenvironment of several malignancies.^42^, ^45^ Infiltration by these cells was negatively correlated with tumor stage, which suggested that CD56^bright^ NK cells might be important for antitumor surveillance.^45^ Interestingly, our results suggest that HRG both increases direct cytotoxic activity and the proportion of CD56^bright^ NK cells. Therefore, it may be possible that HRG exerts antitumor activity by enhancing direct antitumor killing and tumor immunosurveillance. In addition, we showed that HRG increases the secretion of RANTES, which is a chemokine known to regulate the CTL response. Combining these findings with previous research indicating that RANTES enhances antitumor immunity in in vivo models,^46^ we speculate that HRG not only confers NK cell‐mediated antitumor immunity but also induces the migration of CTLs to tumor cells via the activity of RANTES, which are released from NK cells. This hypothesis is supported by the present results confirming that HRG significantly enhances the cytotoxic effects of NK cells toward K562 cells.

PD‐1, a member of the co‐signaling B7 family, plays a role in downregulating NK cell immunity against hematological tumors.^47^ Previous research suggested that PD‐1 expression could be a marker of NK cell functional exhaustion in patients with Kaposi sarcoma.^48^ Further, a recent report indicated that PD‐1 expression on NK cells is crucial for innate immune regulation against tumors.^47^ To our knowledge, no other endogenous factors have been shown to modulate PD‐1 expression on NK cells. Intriguingly, HRG dramatically decreased NK cell surface PD‐1 expression, which was associated with augmented cytotoxic activity against K562 cells, a chronic myelocytic leukemia cell line. Because PD‐1‐positive NK cells are suggested to be anergic, indicating functional exhaustion,^48^ the suppression of a PD‐1 negative checkpoint in NK cells might lead to elevated effector cell functions.

CLEC‐1B, which is expressed diversely on myeloid cells including NK cells and platelets, is involved in various aspects of innate immune responses against infections and tumors.^37^, ^39^, ^40^ Recently, CLEC‐1B expression has received attention for its prognostic value in some types of malignancies; specifically, low CLEC‐1B expression indicates poor prognosis.^37^ Moreover, a previous report suggested that CLEC‐1B might suppress tumor aggressiveness via the phosphoinositide 3‐kinase/AKT pathway.^40^ Based on the previous finding that low CLEC‐1B and high PD‐L1 expression both indicate poor prognosis for malignancies, it is plausible that HRG, which functions via CLEC‐1B to suppress NK cell surface PD‐1 expression has an important role in oncologic immunotherapy.

We also revealed that HRG modulates the nuclear shapes of NK cells, such that they become rounder. Additionally, we discovered that HRG modulates F‐actin distribution by inducing its precise arrangement circumferentially below the cytoplasmic membrane. This change in F‐actin distribution was previously observed in neutrophils,^18^ suggesting that HRG might exert its function on different myeloid cells in a similar fashion. Cell nuclear shapes are partially controlled by cytoskeletal tension, mainly induced by actin.^49^ Rounder cell nuclei reportedly correspond to increased gene expression, which might reflect increased cell activity.^49^ F‐actin remodeling is important for the regulation of cytotoxic granule secretion.^32^, ^44^ Notably, our data provide evidence that HRG also influences actin remodeling, which could establish streamlined cytotoxicity via lytic granule secretion, as well as cytokine production and possibly elevated gene expression.

**Figure 7:**
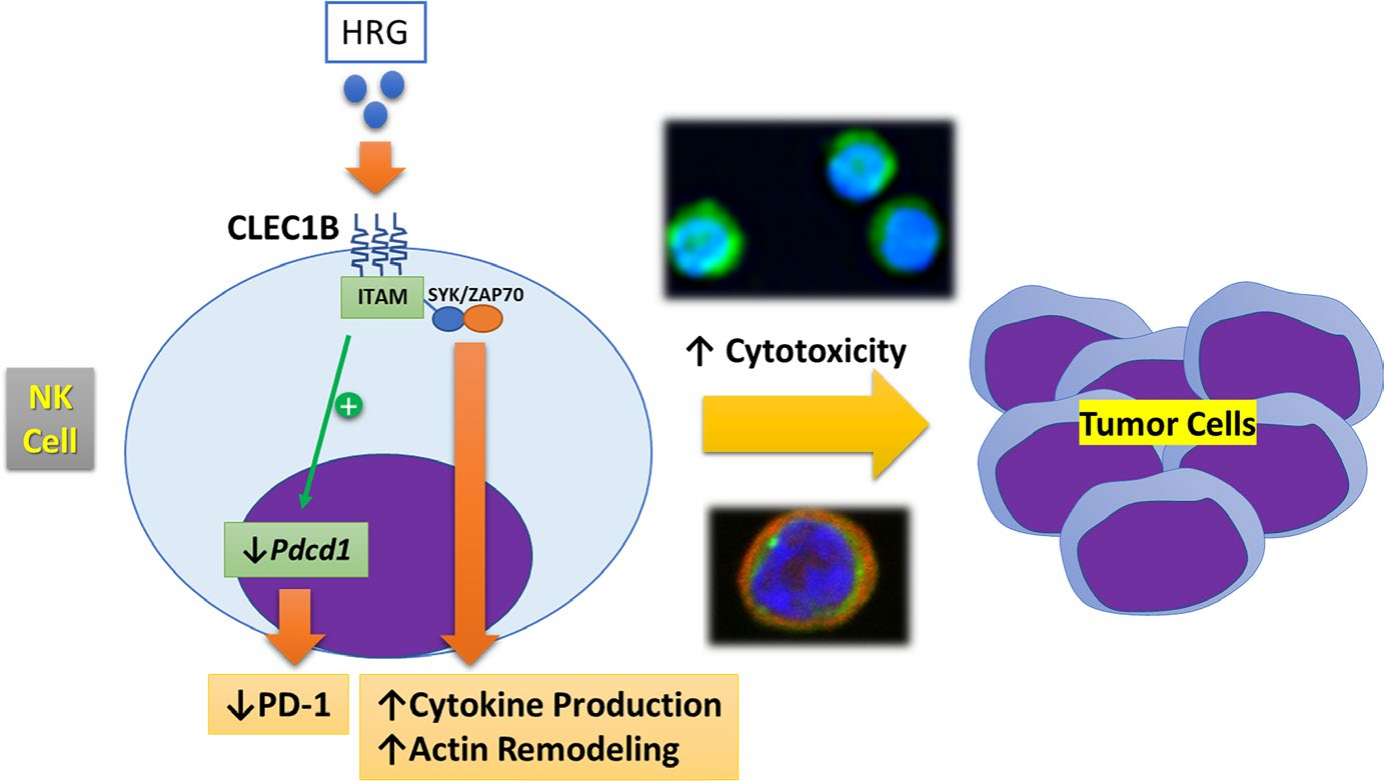
Schematic overview of the hypothesized effects of histidine‐rich glycoprotein (HRG) on natural killer (NK) cells. The hypothesized effects of HRG on NK cells might be mediated by CLEC1B. CLEC1B: C‐type lectin‐like receptor 1B, ITAM: immunoreceptor tyrosine‐based activation motif, *Pdcd1*: programmed cell death 1 gene, PD‐1: programmed cell death protein 1

Overall, our results illuminate the diverse and significant effects of HRG on NK cell cytotoxicity, which was found to occur through various pathways, via CLEC‐1B. Figure 7 is a schematic overview of the hypothesized mechanism through which HRG modulates NK cell functions. Future studies should include: (a) a binding assay of HRG and CLEC‐1B, comparing the binding activity with that of a known ligand, such as podoplanin, (b) a study to investigate whether HRG activates NK cells through not only direct binding to the NK cells but also by influencing the crosstalk between NK cells and K562 cells, (c) a study to clarify the binding affinity of PD‐1 and HRG, and (d) a confirmation of the relationship between the antitumor effects of HRG and NK cell cytotoxicity in in vivo models. The fact that HRG modulates NK cell granzyme B and cytokine production, PD‐1 expression, and morphology indicates its potential as a therapeutic target for antitumor immunity.

## DISCLOSURES

The authors declare no competing financial interests.

## AUTHOR CONTRIBUTIONS

Y.N., H.W., and M.N., designed the research; Y.N. and Y.Y. performed the research; H.W., K.T., D.W., M.S., F.O., and M.N. supervised and edited the manuscript. Y.N. analyzed data and wrote the manuscript.
